# Application of ORF3 Subunit Vaccine for Avian Hepatitis E Virus

**DOI:** 10.3390/vetsci9120676

**Published:** 2022-12-05

**Authors:** Hongjian Yan, Zengna Chi, Hui Zhao, Yawen Zhang, Yuduo Zhang, Yixin Wang, Shuang Chang, Peng Zhao

**Affiliations:** 1 College of Veterinary Medicine, Shandong Agricultural University, Tai’an 271018, China; 2 Shandong Provincial Key Laboratory of Animal Biotechnology and Disease Control and Prevention, Tai’an 271018, China; 3 Shandong Provincial Engineering Technology Research Center of Animal Disease Control and Prevention, Tai’an 271018, China

**Keywords:** avian hepatitis E virus, ORF3, subunit vaccine, prokaryotic expression

## Abstract

**Simple Summary:**

Avian hepatitis E has been widespread in many countries in the last few years and has caused immense economic losses to the poultry industry worldwide. There are still no commercial avian hepatitis E virus (HEV) vaccines so far. ORF3 protein subunit vaccines for HEV strain in laying hens and broilers were prepared, respectively, in this study; and the challenge protection test confirmed that the vaccine could reduce the viral shedding in feces after HEV infection; it provides necessary technical reserve for the prevention and control of avian HEV under existing conditions.

**Abstract:**

Avian hepatitis E virus (HEV) is the main etiologic pathogen of chicken big liver and spleen disease which is widely prevalent in China in recent years. However, due to the lack of a highly effective culture system in vitro, a genetically engineered subunit vaccine is the main direction of vaccine development. In this study, ORF3 genes of VaHEV strain from laying hens and YT-aHEV strain from broilers were amplified, respectively, and ORF3 protein was successfully expressed by *Escherichia coli* prokaryotic expression system. The serum samples were collected periodically to detect avian HEV antibodies by indirect immunofluorescence after specific pathogen free chickens immunized with the two proteins and their mixed proteins, the results showed that all serum samples were positive for antibodies to avian HEV. The antibody-positive chickens were artificially challenged with the cell-adapted strain YT-aHEV strain. The chickens from the immunized control group were infected successfully; no fecal detoxification was detected in the immunized group. In this study, two representative strains of ORF3 subunit vaccines of laying hens and broilers were prepared by prokaryotic expression, the immune effects of different proteins of these were evaluated through immunization and challenge studies in vivo, which provided a new technical possibility for prevention and control of avian HEV.

## 1. Introduction

Avian hepatitis E virus (HEV) is considered to be the major causative agent of big liver and spleen (BLS) disease and hepatic rupture and hemorrhage syndrome (HRHS) in chickens. Avian HEV has caused huge economic losses to the poultry industry worldwide. As early as 2010, it was found that the genome sequence of avian HEV isolates from China had 98.3% homology with European avian HEV [1], and the results of epidemiological investigation in recent years showed that the positive infective rate of avian HEV is increasing in China [2,3,4,5,6,7,8]. There are still no commercial avian HEV vaccines so far.

Developing a vaccine for control of this disease with higher efficacy and fewer side effects is highly desirable, there are no preventive vaccines due to the lack of efficient culture systems that can be used for large-scale culture of avian HEV, which has brought difficulties to the research on inactivated and live attenuated vaccines; therefore, most studies have focused on avian HEV genetic engineering subunit vaccines, Wang et al. used *Lactobacillus lactis* NZ9000 as the delivery carrier of avian HEV immunogenic antigen, selected three truncated regions of ORF2 protein for expression, and found that it could effectively control the avian HEV infection of chickens after oral immunization [9]. The complete genome of avian HEV consists of three open reading frames (ORFs), ORF1, ORF2, and ORF3 [10]. ORF3 encodes the smallest phosphorylated protein, recent studies have revealed that ORF3 contains antigenic epitopes capable of stimulating a strong immune response to the animal organism and can also affect in virus assembly and release of viral particles, immunosuppression, and cell signaling [11], and is considered one of the potential candidate target proteins for the development of avian HEV subunit vaccines. The immune protection of recombinant ORF3 protein was analyzed in young chickens aged 6–8 weeks [11,12]. The results showed that ORF3 protein provided partial immune protection in chickens. The ORF3 gene of the VaHEV strain and the YT strains identified in laying hens and broiler were amplified, respectively, and specific pathogen-free (SPF) chickens were immunized with ORF3 protein as an immunogen. The immunogenicity and protective effect on HEV infection was observed in this study.

## 2. Materials and Methods

### 2.1. Experimental Chicks

Forty-eight 1-day-old SPF chickens were purchased from Dongyue Breeding Birds Co., Ltd (Tai’an, China). Birds were reared on metal cages in separate rooms and fed antibiotic-free standard broilers rations ad libitum with continuous lighting. The animal care and use protocol were approved by the Shandong Agricultural University Animal Care and Use Committee (SDAUA-2016-002). All the experimental animals of this study were cared for and maintained throughout the experiments strictly following the ethics and biosecurity guidelines approved by the Institutional Animal Care and Use Committee of Shandong Agricultural University.

### 2.2. Viruses and Plasmids

An VaHEV strain (GenBank accession no. MG976720.1) was a wild strain identified from layer chicken flocks in Hebei Province, China; the whole genome plasmid was constructed by Su et al. [4]; an YT-aHEV strain (GenBank accession no. MZ736614.1) was a wild strain isolated and identified from broiler breeder flocks in Shandong Province, China. The whole-genome plasmid was constructed by Zhang et al. [6]. Due to the VaHEV strain being unable to replicate in cell culture (while the YT-aHEV strain can), the YT-aHEV strain was selected for the virus challenge protection test.

### 2.3. Primer Designs and Synthesis

Based on the nucleotide sequence of YT-aHEV strain and VaHEV strain, specific primers were designed by Primer 5.0 (Primer Premier, freeware) to amplify the whole ORF3 gene, restriction enzyme cutting site of *BamHI* and *XhoI* were introduced into the two ends of each primer. All the primers were synthesized by Sangon Biotech (Shanghai) Co., Ltd., Shanghai, China and are listed in Table 1.

### 2.4. Amplification of ORF3 Genes and Plasmids Construction

The whole-genome plasmids of VaHEV and YT-aHEV strains were used as the templates to amplify the ORF3 genes of both strains with the primers listed in Table 1, respectively. The PCR mixtures were prepared in 25 μL volumes containing 10 × buffer 2.5 μL, Taq enzyme 0.5 μL, primer 1μL, H_2_O 17μL. The PCR reaction was performed under the following conditions: pre-denaturation at 95 °C for 5 min, then 34 cycles of denaturation at 95 °C for 30 s, annealing at 58 °C for 30 s, extension at 72 °C for 30 s, and a final extension at 72 °C for 10 min. The PCR products were subjected to 1% agarose gel electrophoresis, purified by a gel DNA Recovery Kit (TianGen, Beijing, China) in accordance with the manufacturer’s protocol and ligated to pMD™ 18-T vector (Takara, Beijing, China) for sequencing validation, the validated plasmid was double-digested with pEASY-Blunt (TransGen, Beijing, China) expression vector by *XhoI* and *BamHI* restriction enzymes. The digested product was ligated by T4 DNA Ligase Kit to construct recombinant expression plasmid. The sequenced expression plasmids were named YT-HEV-ORF3 and VaHEV-ORF3, respectively.

### 2.5. Protein-Induced Expression and Western Blot Identification

*Escherichia coli* strain carrying YT-HEV-ORF3 and VaHEV-ORF3 plasmids was cultured in 200 mL LB broth containing 100 μg/mL ampicillin and incubated at 37 °C for 2–3 h with 220 rpm shaking until OD_600_ reached up to 0.8. The culture was induced by 1mmol/L (final concentration) IPTG and further incubated at 30 °C for 6 h. The bacteria were disrupted by cycles of sonication and freeze thaw. Supernatant and pellet (inclusion bodies) were separated by centrifugation at 12,000 rpm for 2 min at 4 °C, the centrifuge retained precipitation and dissolved 40 μL of lysate and 10 μL of 5× sampling buffer mix together and boiled for 6 min, carrying out accelerated cooling at −20 °C and 10% SDS-PAGE electrophoresis was used to analyze the protein expression. The target protein was loaded to the chromatographic columns having Ni NTA resin and allowed to pass through slowly to ensure maximum binding, and the purified protein was identified and analyzed by 10% SDS-PAGE electrophoresis, then transferred onto PVDF membrane. The membranes were then blocked with 5% powder skim milk for 2 h, incubated with mouse-derived HIS primary antibodies (Abcam, UK) against the targeted protein overnight at 4 °C; after washing in TBST (160 mM Tris-buffered saline with 0.05% of Tween 20) each membrane was incubated with the anti-mouse secondary antibody (Abcam, UK) for 2 h at room temperature for color development.

### 2.6. Vaccine Immunization

**Figure 1:**
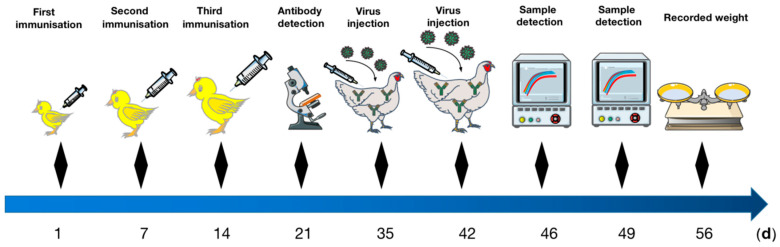
Diagram of the experimental immunization procedure in animals.

Proteins quantification were carried out using a trace BCA protein quantification kit. The quantified protein was diluted to 1000 μg/mL, mixed with the same amount of adjuvant, the mixture was emulsified by an electric stirrer and stored at 4 °C for use. Forty-eight 1-day-old SPF chickens were randomly divided into four groups. Chickens of Group 1 were immunized with YT-aHEV protein, Group 2 were immunized with VaHEV protein, group 3 were immunized with a mixture of YT-aHEV and VaHEV protein, and the control group were administered with normal saline. Up to 0.2 mL emulsion containing 100 μg of target protein was subcutaneously injected into the neck of the chicken for immunization. After the first immunization at 1 day old, the immunization was carried out twice, a total of three times, each time with an interval of one week. Blood samples were collected one week after each immunization (Figure 1).

### 2.7. IFA Detections of HEV Antibodies

The YT strain was inoculated into leghorn male hepatocellular cells (LMH, this cell line was purchased from ATCC CRL-2117). The cells were washed three times with PBS after the cells grew into monolayer and fixed with 4% paraformaldehyde overnight at 4 °C. The cells were then washed again with PBS buffer and the chickens sera of four treatment groups were used for 50× dilution and inoculated into LMH as the first antibody at 37 °C for 1 h. After incubation, the cells were washed again with sterile PBS, the FITC-labelled rabbit anti-chicken IgG antibody was added as a secondary antibody and the samples were then protected from light and incubated at 37 °C for 1 h. After incubation, the cells were washed again with PBS and infected cells were examined under a fluorescent microscope. To compare the antibody levels after immunization with both YT-ORF3 and Va-ORF3 proteins, two chickens’ sera were diluted in dilution buffer at 1:50, 1:100, and 1:150 and used as primary antibodies for IFA according to the above procedure.

### 2.8. Artificial Challenge Test

To detect the chickens were positive for HEV antibodies after immunization with protein, each immune group and control group chickens were challenged with avian HEV for two tests at 35 and 42 days of age. Each chicken was inoculated with a total of 600 TCID_50_ YT-aHEV strain by intravenous injection and intramuscular injection, and each method was injected with 300 TCID_50_. On the fourth and seventh days after all chickens were challenged, six chickens from each group were randomly selected to collect cloacal swabs for detection. After RNA extraction from cloacal swabs samples, the avian HEV viral load was detected by real-time fluorescence quantitative PCR (RT-qPCR) according to the published method [13]. The cycle threshold value (Ct-value) less than 30 was defined as a positive. Statistical analysis was performed using the GraphPad Prism 8.0 statistical software package for Windows version 10. *p* < 0.05 was considered statistically significant based on a T-test. Two weeks after inoculation, eight chickens were randomly selected from different immunized groups and weigh the chickens and recorded with an electronic balance. The sera of the non-immune challenge control group after the challenge were tested for antibodies against HEV according to step 2.7, and the successful infection was confirmed in this way.

## 3. Results

### 3.1. Construction and Homology Comparison of the ORF3 Recombinant Plasmids of YT-aHEV and VaHEV Strains

**Figure 2:**
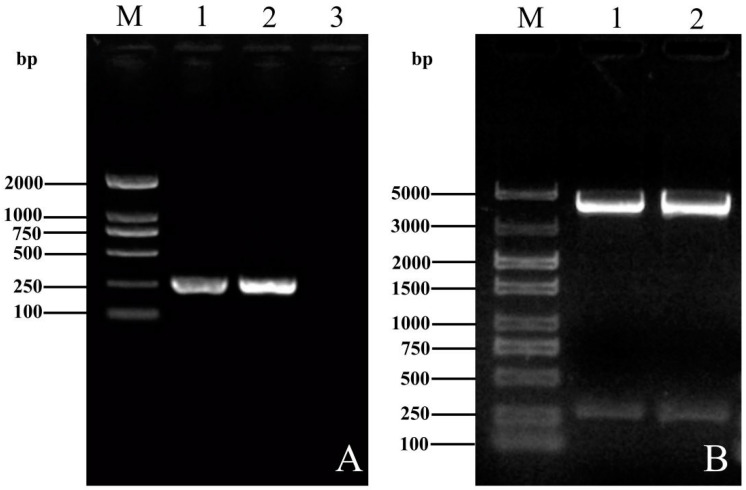
ORF3 plasmid construction. (**A**) Results of ORF3 gene amplification in YT strain and Va-HEV strain. M: DNA 2000 marker; 1: VaHEV ORF3; 2: YT-aHEV ORF3; 3: Negative control. (**B**) Identification of YT-ORF3 and VaHEV-ORF3 recombinant plasmids by enzymatic digestion. M: DNA 5000 marker; 1: VaHEV ORF3; 2: YT-aHEV ORF3.

The amplified PCR products were analyzed by 1% agarose gel electrophoresis and the target gene band at a length of 260 bp was observed (Figure 2A and Supplementary Figure S2). The results of gel electrophoresis showed that the PCR amplification products and the pEASY-Blunt vector at a length of 260 bp and 3929 bp were observed by double digestion with *XhoI* and *BamHI* (Figure 2B and Supplementary Figure S3). The sequencing showed that the ORF3 recombinant plasmids was consistent with the gene sequence of both YT-aHEV and VaHEV strains, the homology of nucleic acid and amino acid of the two strains was 85.1% and 67.8%, indicating that there are differences in ORF3.

### 3.2. Induced Expression, Protein Purification and Western Blot Assay of Recombinant Bacteria

**Figure 3:**
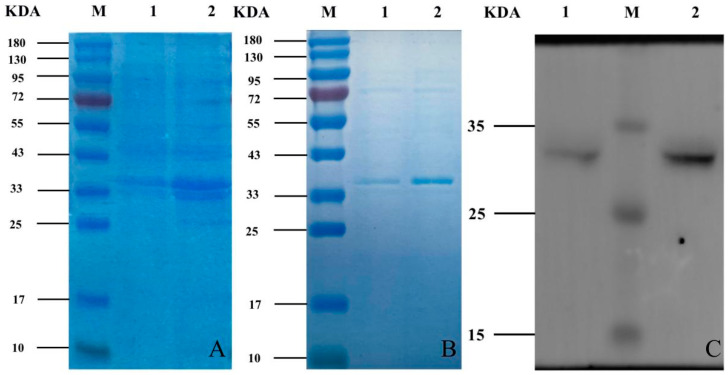
Induced expression, protein purification and western blot assay of recombinant bacteria. (**A**) SDS-PAGE of recombinant bacteria-induced expression of proteins. M: protein molecular weight Marker; 1: YT-aHEV ORF3; 2: VaHEV ORF3. (**B**) SDS-PAGE electrophoresis of purified proteins. M: protein molecular weight standards; 1: YT-aHEV ORF3; 2: VaHEV ORF3. (**C**) Western blotting analysis of recombinant proteins. M: protein molecular weight Marker; 1: YT-aHEV ORF3; 2: VaHEV ORF3.

After sonication and centrifugation, the supernatant and precipitate were obtained. Both the supernatant and the precipitate were subjected to SDS-PAGE electrophoresis, since HIS tag in the vector is about 14KDa after expression, target protein is about 20KDa, so a band matching the size of the target protein appeared at around 34 kDa (Figure 3A and Supplementary Figure S4). The target protein was expressed mainly existed in the bacteriophage, indicating that the protein was expressed mainly in the form of inclusion bodies.

An obvious target bands of purified protein was shown on the SDS-PAGE electrophoresis (Figure 3B and Supplementary Figure S5). The Western blotting analysis (Figure 3C and Supplementary Figure S1) showed the specific immunoblot bands appeared at 34KDa which was consistent with the expected results. The protein concentration was determined using the BCA protein quantification method; the results showed that the protein concentration of 2147.21 μg/mL for YT-ORF3 and 2454.41 μg/mL for Va-ORF3. The protein was diluted to 1000 μg/mL after quantification and 100 μg per chicken was immunized, the subunit vaccine was prepared with the protein and vaccine adjuvant mix in a ratio of equal volume.

### 3.3. HEV Antibodies Detection by IFA

**Figure 4:**
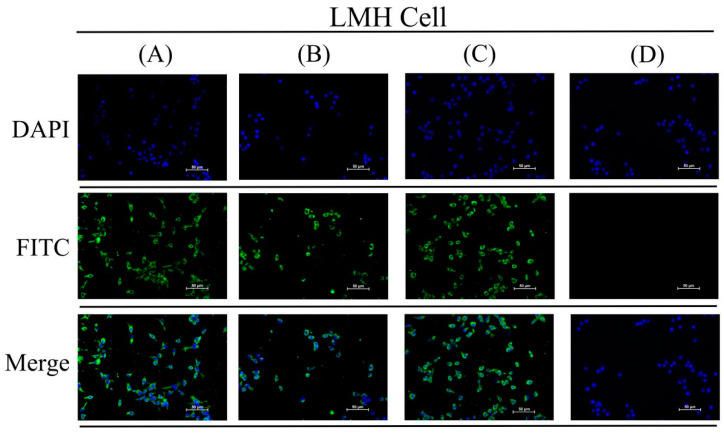
Indirect immunofluorescence of chicken ORF3 antibody serum. (**A**) YT-aHEV; (**B**) VaHEV; (**C**) YT+VaHEV; (**D**) Negative control.

**Figure 5:**
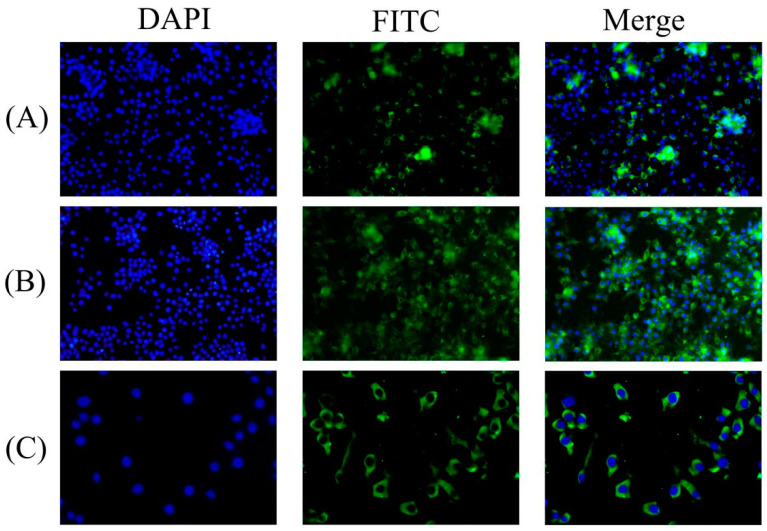
Indirect immunofluorescence of chicken serum. (**A**) Indirect immunofluorescence of VaHEV ORF3 antibody serum after 150-fold dilution. (**B**) Indirect immunofluorescence of YT-aHEV ORF3 antibody serum after 150-fold dilution. (**C**) Indirect immunofluorescence detection of ORF3 antibody sera from unimmunized controls chickens.

The IFA results display that chicken sera from the VaHEV ORF3 protein immunized group, the YT-aHEV ORF3 protein immunized group, and two proteins mixed immunized group were HEV antibody positive. The results indicated that HEV antibody was successfully induced after immunization (Figure 4). Chicken sera were diluted at 1:50, 1:100, and 1:150 from immunized with two ORF3 proteins, respectively, as shown in Figure 5. The serum recognition effect of the YT-aHEV ORF3 protein immunized group was better than the VaHEV ORF3 protein immunized group when diluted at 1:150.

### 3.4. Results of the Challenge Experiment

**Figure 6:**
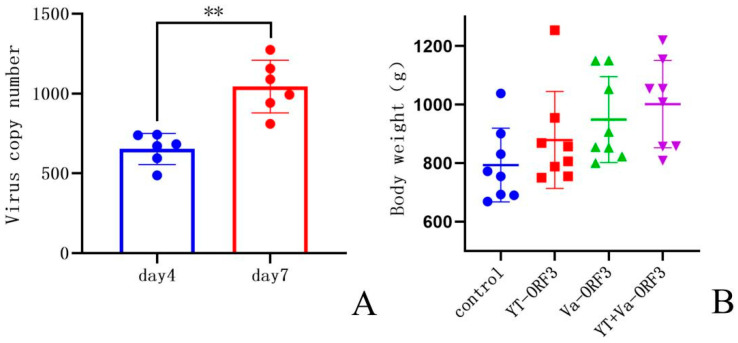
Virus copy numbers for the control group and body weight for each experimental group. (**A**) Virus copy numbers on day 4 and 7 in the non-immunized control group, **: *p* < 0.01. (**B**) The body weight of chickens in the control and different immunization groups.

The sample of cloacal swabs from each treatment group were determined by RT-qPCR against HEV after twice challenged with the YT strains. The results showed that the positive rate of avian HEV nucleic acid in cloacal swabs from the non-immunized control group was 100% after 4 days and 7 days post-infection, while the positive rate of avian HEV nucleic acid in cloacal swabs from the YT-aHEV protein immunized group and the mixed protein immunized group was 0. The positive rate of avian HEV nucleic acid in cloacal swabs from the VaHEV protein immunized group was 20% after 4 days post-infection and decreased to 0 after 7 days post-infection (Table 2). This indicates that the antibodies by immunization blocked the proliferation and shedding of avian HEV in vivo successfully. The IFA test results were determined avian HEV antibody positive, when the chicken serum of the unimmunized control group was used as the first antibody (Figure 5C), indicating that HEV infection has achieved good results. The results indicated that the copy number of avian HEV from the cloacal swabs samples of the unimmunized protein control group on days 4 and 7 showed a significant upward trend in viral shedding (*p* < 0.01) (Figure 6A and Supplementary Table S1). After two weeks post-infection, eight chickens in each group were randomly selected and weighed, the results indicated that avian HEV infection would induce growth inhibition in chickens, and ORF3 subunit vaccine could reduce the adverse effect of avian HEV on production performance (Figure 6B and Supplementary Table S2).

## 4. Discussion

Avian HEV is thought to be the main causative agent of big liver and spleen (BLS) disease, hepatic rupture and hemorrhage syndrome (HRHS) in chickens. Infected chickens show pathological symptoms such as hepatosplenomegaly and hemoperitoneum, hepatic steatosis and amyloidosis, which cause serious harm to chicken flocks. It has been reported that subgroup J avian leukosis virus and avian HEV co-infection were detected in chickens with HRHS [14]. There are other reports which found that the egg production in laying hens decreased after avian HEV infection [5]. Since the first case of avian HEV was discovered, avian HEV epidemics have been identified in many countries around the world [2,3,4,5,6,7,8,15,16,17,18,19,20,21,22].

Due to the lack of an effective cell culture system for avian HEV, there is no effective inactivated whole virus vaccine or live attenuated vaccine available for the prevention and control of the disease; the mechanisms of HEV pathogenesis are still not well understood. Some strategies have been explored in attempts to produce effective vaccine such as subunit vaccine [9,23,24,25,26]. The development of subunit vaccine is one of the main approaches that are expected to break through at present. The avian HEV genome size is approximately 6.6 kb, three open reading frames in HEV genome, with ORF3 and ORF1 not overlapping, which is a slight difference between avian and mammalian HEV. The ORF2 protein contains multiple antigenic epitopes and is often the focus of vaccine research. The chickens were immunized with recombinant ORF2 protein and challenged with HEV, the results confirmed that the recombinant ORF2 protein provided only partial immune protection to experimental chickens [27]. In addition, the six antigenic epitopes of the ORF2 protein have been analyzed several times and it was found that some of them are not very promising for vaccine development [28].

The ORF3 protein is a phosphoprotein encoded by the third ORF of HEV, ORF3, and the function of ORF3 has been poorly studied. Several studies have speculated that ORF3 protein may be part of the HEV envelope protein and is associated with viral pathogenicity [29,30,31,32]. In recent years, it has been found that ORF3 protein plays a key role in virus assembly and release of viral particles, cell signaling, immunosuppression, and cytoskeleton assembly. Recent studies have focused on the potential of ORF3 protein as an immunogen; it has been demonstrated that ORF3 protein can also induced immune responses to produce IgM antibodies [33]. In this study, the ORF3 proteins of YT-aHEV and VaHEV were successfully expressed using a prokaryotic expression system, and these gene regions are thought to be the core regions that may play a major role in signaling and are relatively conserved in the virus [11]. The expressed proteins all produced high titer levels of avian HEV antibody after immunization in chickens, indicating the expressed proteins have strong immunological activity. Previous studies have analyzed the immune protection in the ORF3 protein of avian HEV and found that the ORF3 protein provided a partial immune protection to experimental chickens, and also can delay and reduce the viral shedding in feces of infected chickens [11]. The results of this study showed that the three ORF3 protein subunit vaccines prepared had an important blocking effect to reduce viral shedding in feces after HEV infection. Kenney et al. has found that ORF3 protein expression prompted the release of infectious viral particles from host cells and therefore the infectivity of chicken to avian HEV could decreased after ORF3 protein immunization in chickens [34], which is also consistent with the results of this study. The viral shedding in feces is one of the main modes of HEV transmission, and vaccination can significantly reduce or even block viral shedding in feces of avian HEV, thus, may be promising as a potential vaccine candidate against avian HEV infection. It has been shown that ORF2 protein mainly induced immune responses to produce IgG antibodies, whereas ORF3 protein induced IgM antibodies [33]. Since IgM mainly appears at the early stage of infection, it is assumed that ORF3 protein immunization may play a more important role than ORF2 protein in blocking early HEV infection.

Another major factor that must be considered in the preparation of subunit vaccines is the high variability of avian HEV. Su has analyzed the sequencing of the ORF2 gene from 78 positive samples in China, the sequence homology of 78 ORF2 gene ranged from 74.4% to 100% within a large variation, exhibiting a high degree of genetic diversity of avian HEV in China [4]. The ORF3 nucleic acid and amino acid homology of YT-aHEV and VaHEV strains were also compared in this study, it was found that there were significant differences between the two strains. Although the two ORF3 proteins are quite different, both the ORF3 protein of the laying hen-derived VaHEV strain and the broiler-derived YT-aHEV strain provided good protection against YT-aHEV strain infection after immunization, and it was speculated that the ORF3 fragments of the two strains may share a common dominant antigenic epitopes, which also confirmed that the results of previous studies on ORF3 antigen epitope identification [26]. However, the YT-aHEV strain for ORF3 protein provided better immune effect than that of VaHEV strain for ORF3 protein at 4 days after inoculation, this indicated that there was significant difference in antigenicity. Therefore, two different ORF3 protein combinations were creatively immunized with chickens which undoubtedly improved the immune protection range of ORF3 protein subunit vaccine. Vaccine prepared in this study are going to carry out clinical observation in a wider range. It is worth noting that with the variation of the virus, we need to consider the use of different serotypes of protein for a mixed preparation of vaccines, which also indirectly increases the cost of vaccines, but compared with the relatively high-cost vaccines such as adenovirus subunit vaccine and infectious bursal disease virus-like particle vaccine used in commercial chickens, the ORF3 subunit vaccine we use in breeding chickens shows the advantage of low cost.

## 5. Conclusions

Overall, subunit vaccine was prepared according to the ORF3 gene sequence of laying hen HEV strain and broiler HEV strain. The challenge protection test of YT-aHEV strain confirmed that the prepared vaccine could reduce the viral shedding in feces after HEV infection. In particular, the effect of broiler ORF3 protein was better, providing a necessary technical reserve for the prevention and control of avian HEV under existing conditions.

## Figures and Tables

**Table 1:** Primer information for amplification of ORF3 gene.

Primer Name	Primer Sequences (5′–3′)
Va-ORF3-F	GGATCCatgCGCCTCGGCTGCCAGCAC (*BamHI GGATCC*)
Va-ORF3-R	CTCGAGctaCATCTGGTACCGTGCG (*XhoI CTCGAG*)
YT-ORF3-F	GGATCCatgTGTCTTAGTTGCCAGTT (*BamHI GGATCC*)
YT-ORF3-R	CTCGAGctaCGTCTGGTACCGTGCGA (*XhoI CTCGAG*)

Small letters “atg” and “cta” show the initiation and the complementary sequence of termination codons, respectively.

**Table 2:** Results of positive detection rate of anal swab after challenge.

Days After Challenge	VaHEV Protein Immunization Group	YT aHEV Protein Immunization Group	Mixed Immunization Group	Negative Control Group
2	5/8	5/8	3/8	8/8
4	1/8	0/8	0/8	8/8
7	0/8	0/8	0/8	8/8

## Data Availability

Data are contained within the article or supplementary material.

## Supplementary Materials

The following supporting information can be downloaded at: https://www.mdpi.com/article/10.3390/vetsci9120676/s1. Figure S1: Western blotting analysis of recombinant proteins. Figure S2: Results of ORF3 gene amplification in YT strain and Va-HEV strain. Figure S3: Identification of YT-ORF3 and VaHEV-ORF3 recombinant plasmids by enzymatic digestion. Figure S4: SDS-PAGE of recombinant bacteria-induced expression of proteins. Figure S5: SDS-PAGE electrophoresis of purified proteins. Table S1: Virus copy numbers on day 4 and 7 in the non-immunized control group. Table S2: The body weight of chickens in the control and different immunization groups.
