# Supplementary material for: Application of ORF3 Subunit Vaccine for Avian Hepatitis E Virus
Source: Vet Sci. 2022 Dec 5;9(12):676. doi: 10.3390/vetsci9120676 (PMC9784926; doi:10.3390/vetsci9120676)
Supplement: Supplementary file 1 [file vetsci-09-00676-s001.zip › vetsci-2011609-supplementary.pdf]

Table S1 Virus copy numbers on day 4 and 7 in the non-immunized control group

	CT value of the day 4	Number of virus copies on day 4	CT value of the day 7	Number of virus copies on day 7
1	27.61284293	743.06	26.97497057	1157.31
2	27.93202802	595.3	26.83599601	1274.6
3	27.61901725	739.88	27.06251662	1089.03
4	28.21901291	487.71	27.48699328	810.94
5	27.75753217	672.01	27.27205243	941.52
6	27.73333593	683.4	27.19438395	993.71

x=(y-37.13)/(-3.3149); Notes: y(CT value); POWER(10,x)(Number of virus copies)

Table S2 The body weight of chickens in the control and different immunization groups

control	YT-ORF3	Va-ORF3	YT+Va-ORF3
772	1254	800	858
693	955	1150	1054
1038	869	1053	1155
831	806	823	1008
901	857	855	1054
690	755	907	809
669	750	853	857
755	788	1151	1220

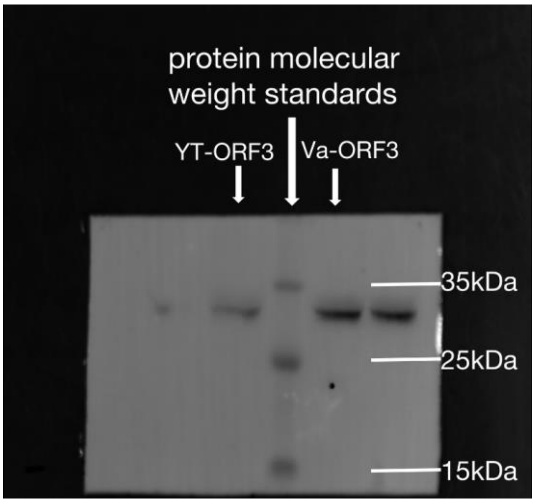

Figure S1 Western blotting analysis of recombinant proteins

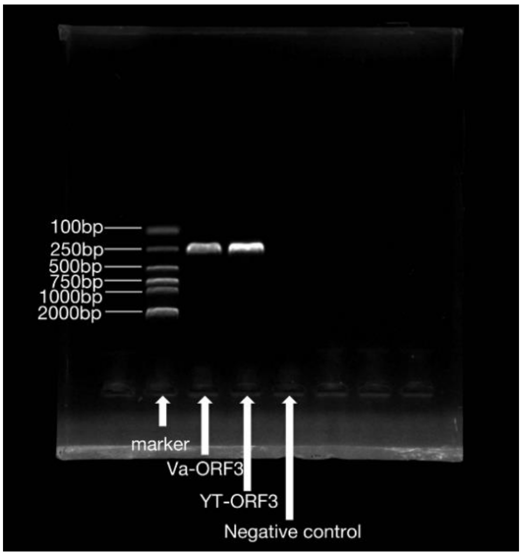

Figure S2 Results of ORF3 gene amplification in YT strain and Va-HEV strain

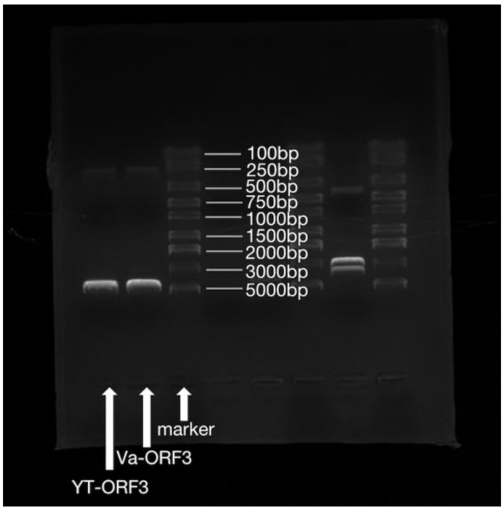

Figure S3 Identification of YT-ORF3 and VaHEV-ORF3 recombinant plasmids by enzymatic digestion

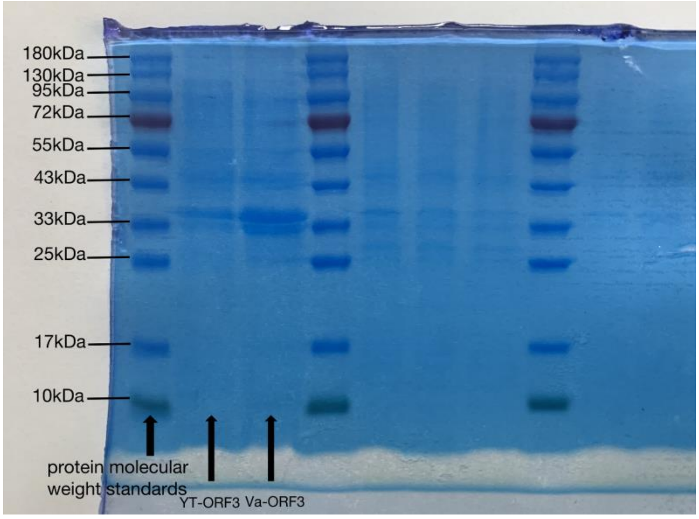

Figure S4 SDS-PAGE of recombinant bacteria-induced expression of proteins

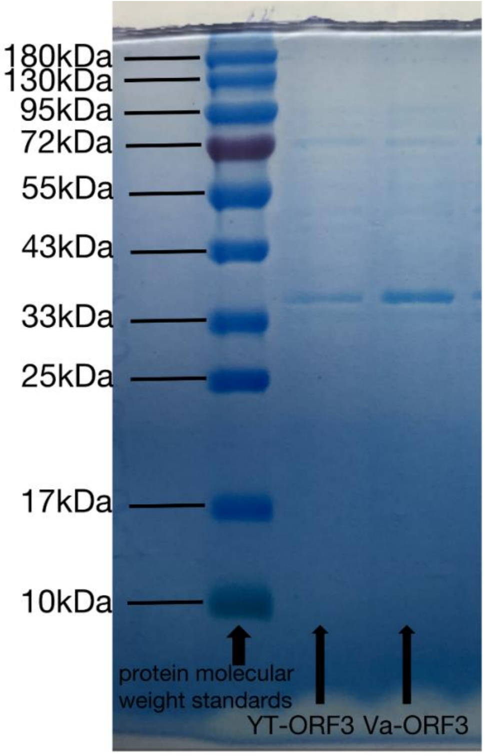

Figure S5 SDS-PAGE electrophoresis of purified proteins
